# COMPARING SUCCESSFUL AGING OF NEAR-CENTENARIANS AND CENTENARIANS: FINDINGS FROM 2011 AND 2021/22

**DOI:** 10.1093/geroni/igac059.2722

**Published:** 2022-12-20

**Authors:** Eric Ngai Yin Shum, Bobo Hi Po Lau, Karen Siu Lan Cheung, Grace Man Yee Chan, Joseph Shiu Kwong Kwan, James Ka Hay Luk, Peter Martin, Cecilia Lai Wan Chan

**Affiliations:** Hong Kong Shue Yan University, Hong Kong, Hong Kong; Hong Kong Shue Yan University, Hong Kong, Hong Kong; The University of Hong Kong, Hong Kong, Hong Kong; The Hong Kong Council of Service, Hong Kong, Hong Kong; Imperial College London, London, England, United Kingdom; TWGHs Fung Yiu King Hospital (FYKH), Hospital Authority, Hong Kong, Hong Kong; Iowa State University, Ames, Iowa, United States; The University of Hong Kong, Hong Kong, Hong Kong

## Abstract

Successful aging (SA) was proposed by Robert J Havighurst in 1961 to capture how older adults add “lives onto (their) years.” While there is a consensus regarding the multidimensionality of the concept, the set of criteria that should be applied to older adults of advanced age remain controversial. Notwithstanding their inevitable decline in physical health, adults of advanced age may still enjoy good psychosocial well-being. In this light, we compared the proportion of “successful agers” in two cohorts of adults aged 95 or above who lived with their families in 2011 (Nf77) and 2021/22 (Nf120) in Hong Kong using two models – Model A: i: Good subjective health, ii: more well-off than average, iii: as happy as young (Cho et al., 2012) and Model B: i: Weekly social activities, ii: absence of dementia, iii: intact sight and hearing ability, iv: intact mobility (Nosraty et al, 2012). Both models have been applied in adults aged 90 or above. In the 2011 cohort, 13.0% and 16.9% of our sample fulfilled the SA criteria of Model A & B respectively. The percentages fell to 1.7% and 13.7% respectively in the 2021/22 cohort. The decrease is due to less participants fulfilling the financial criterion of Model A, as well as the criteria on intact sight and hearing ability and the absence of dementia of Model B. COVID presents multidimensional challenges for adults of advanced age. Examining the dimensions that are most impacted will help orient recovery works along the direction of SA.

